# Genetics of Type 2 Diabetes in African Americans

**DOI:** 10.1007/s11892-015-0651-0

**Published:** 2015-08-19

**Authors:** Maggie C. Y. Ng

**Affiliations:** Center for Genomics and Personalized Medicine Research, Wake Forest School of Medicine, Winston-Salem, NC USA; Center for Diabetes Research, Wake Forest School of Medicine, Winston-Salem, NC USA

**Keywords:** Admixture mapping, African Americans, Fine mapping, Genome-wide association study, Glucose homeostasis, Linkage, Meta-analysis, Population differentiation, Type 2 diabetes

## Abstract

Type 2 diabetes (T2D) is a global health problem showing substantial ethnic disparity in disease prevalence. African Americans have one of the highest prevalence of T2D in the USA but little is known about their genetic risks. This review summarizes the findings of genetic regions and loci associated with T2D and related glycemic traits using linkage, admixture, and association approaches in populations of African ancestry. In particular, findings from genome-wide association and exome chip studies suggest the presence of both ancestry-specific and shared loci for T2D and glycemic traits. Among the European-identified loci that are transferable to individuals of African ancestry, allelic heterogeneity as well as differential linkage disequilibrium and risk allele frequencies pose challenges and opportunities for fine mapping and identification of causal variant(s) by trans-ancestry meta-analysis. More genetic research is needed in African ancestry populations including the next-generation sequencing to improve the understanding of genetic architecture of T2D.

## Introduction

Diabetes is a global health problem affecting 347 million people worldwide [1] and is predicted to be the seventh leading cause of death by 2030 [2]. In the USA, 28.9 million adults have diabetes and 86 million adults have pre-diabetes, accounting for nearly half of the population. Diabetes disproportionally affects the minority populations with higher prevalence in Native Americans (15.9 %), non-Hispanic blacks (13.2 %), Hispanics (12.8 %), and Asian Americans (9 %) as compared to non-Hispanic whites (7.6 %) [3]. Among all diabetes cases, about 90 % are classified as having type 2 diabetes (T2D). T2D is characterized by hyperglycemia resulting from both pancreatic beta cell dysfunction with decreased insulin secretion and insulin resistance at target tissues. T2D susceptibility is strongly influenced by aging, obesity, family history, and ethnicity. In concordance with the observations in adults, the prevalence of T2D is substantially higher in children of minority populations than in non-Hispanic whites (0.034–0.12 % vs. 0.017 %, respectively) while the reverse trend is observed for type 1 diabetes (0.035–0.162 % vs. 0.255 %, respectively) [4]. The disparity of T2D in the minority populations is partly contributed by the mirroring prevalence of obesity in non-Hispanic blacks (47.8 %), Hispanics (42.5 %), and non-Hispanic whites (32.6 %) [5]. In addition, T2D-associated comorbidities including kidney disease, retinopathy, and cardiovascular diseases are also highly prevalent in minority populations [6–12]. Diabetes is a huge burden to public health, with direct medical and indirect costs totaling $245 billion [3].

T2D and associated complications have been attributed to behavioral and socio-economic factors [13–15] but are also influenced by genetic factors as evidenced by strong clustering of diabetes within families [16–18]. Currently, most genetic research on T2D is being conducted in European ancestry populations. Little is known of whether individual genetic background contributes to racial disparity of T2D in minority populations. This article will review the genetic research of T2D and respective glycemic traits conducted in populations of African ancestry and highlight their importance in understanding the genetic risk factors predispose to T2D.

### Linkage Mapping

Several genome-wide linkage studies in families of African ancestry have been conducted to search for chromosomal regions linked to T2D and glycemic traits. The Africa America Diabetes Mellitus (AADM) study recruited 343 T2D-affected sibling pairs from West Africa. Evidence of suggestive linkage for T2D was found on chromosomes 12q, 20q, and 19p, of which the former two linkage peaks had been reported in other populations [19]. In addition, four genetic loci at chromosomes 10q, 4p, 15q, and 18p were linked to C-peptide concentration [20]. The Sea Islands Genetic African American Registry studied 521 Gullah-speaking African Americans from 197 families and identified suggestive linkage at chromosomes 14q, 1q, 3q, and several regions at 7p [21]. The Family Investigation of Nephropathy and Diabetes (FIND) study included 1004 African Americans from 362 families and identified significant linkage at chromosome 4q, and suggestive linkage at chromosomes 12q and 22q [22]. The largest T2D linkage analysis in African Americans was conducted by the Genetics of Non-Insulin-Dependent Diabetes Mellitus (GENNID) study and included 1496 individuals from 580 African American families. Significant and suggestive linkage for T2D was identified on chromosomes 2p, and 13p and 7p, respectively [23]. Subsequent fine mapping suggested the presence of multiple susceptibility genes under the linkage peaks, including strong association at *GRB10* on chromosome 7p [24]. It is intriguing that the *GRB10* locus was associated with fasting glucose level in European populations [25] but showed no evidence of association for T2D in African Americans [26••]. Collectively, despite the presence of suggestive evidence of linkage at multiple chromosomal regions, none overlapped among the African ancestry studies, and the causal genes underlying the linkage peaks, if any, have not been identified. The lack of strong association of common variants suggests rare variants with strong effect may account for some of the linkages. The challenge of mapping T2D loci by linkage highlights the complex genetic architecture of T2D [27].

### Genome-Wide Association Mapping

Genome-wide association studies (GWAS) have successfully identified common variants in thousands of genetic loci associated with complex traits in the past decade. The first series of T2D GWAS conducted in 2007 in populations of European ancestry identified eight loci at or near *TCF7L2*, *SLC30A8*, *HHEX*, *CDKAL1*, *CDKN2A/B*, *IGF2BP2*, *KCNJ11*, and *FTO* [28–31]. Subsequently, rapid discovery has been facilitated by increased density of genotyping arrays, improvement of haplotype reference panels and imputation methods, and most importantly, increased sample size through international collaborations. The DIAbetes Genetics Replication And Meta-analysis (DIAGRAM) consortium and the Meta-Analyses of Glucose and Insulin-related traits consortium (MAGIC) conducted large-scale meta-analysis of GWAS for T2D and glycemic traits, respectively, in European ancestry populations. Up to now, they and others have identified >80 loci associated with T2D, and >70 loci associated with glycemic traits including fasting glucose, fasting insulin, fasting proinsulin, insulin secretion and insulin resistance measured by homeostatic model assessment (HOMA), glucose measured at oral glucose tolerance test, corrected insulin response to glucose, and HbA_1c_ [32•, 33].

The majority of loci for T2D and glycemic traits have been identified from European ancestry populations [34, 35]. It is unclear whether genetic variants and loci identified from European ancestry populations are transferable to non-European populations, and if ancestry-specific genetic loci are present. Early GWAS in non-European populations had relatively small sample sizes that limit detection of common genetic variants with moderate effects [36–38]. The first T2D GWAS in African Americans was reported in 2012 and identified a novel locus near *RND3* and *RBM43* from 1994 discovery and 4455 replication/validation samples [39•]. Another GWAS that examined fasting insulin and insulin resistance from the HOMA model in 1497 African American and West African subjects identified two novel loci at *SC4MOL* and *TCERG1L* [40•] (Table 1).

**Table 1:** Genome-wide significant loci associated with T2D and glycemic traits in African ancestry populations

Trait	Locus	SNP	OR/beta (95 % CI)^a^	Initial sample size (total or case/control)	Replication sample size (total or case/control)	Reference
T2D	*RND3-RBM43*	rs7560163	0.75 (0.67–0.84)	965/1029	2167/2288	[39]
T2D	*TCF7L2*	rs7903146	1.36 (1.32–1.4)	8284/15,543	14,191/44,470	[26••]
T2D	*KCNQ1*	rs231356	1.09 (1.06–1.13)	8284/15,543	14,191/44,470	[26••]
T2D	*KCNQ1*	rs2283228	1.19 (1.13–1.24)	8284/15,543	14,191/44,470	[26••]
T2D	*HMGA2*	rs343092	1.14 (1.1–1.19)	8284/15,543	14,191/44,470	[26••]
T2D	*HLA-B*	rs2244020	1.09 (1.06–1.13)	8284/15,543	14,191/44,470	[26••]
T2D	*INS-IGF2*	rs3842770	1.14 (1.09–1.19)	8284/15,543	6061/5483	[26••]
Fasting insulin	*SC4MOL*	rs17046216	0.18 (0.12–0.24)	927	570	[40•]
Fasting insulin	*TCERG1L*	rs7077836	0.28 (0.18–0.38)	927	570	[40•]

^a^Odds ratio are presented for T2D and effect size are presented for glycemic traits under additive model

The Meta-analysis of the Type 2 Diabetes in African Americans (MEDIA) consortium was formed with the aim of improving the power of GWAS to identify T2D loci in African Americans. A similar effort for meta-analysis of GWAS for glycemic traits is currently underway by the African American Genetics of Glucose and Insulin (AAGILE) consortium. The MEDIA consortium has recently conducted a large-scale meta-analysis of T2D GWAS that included 23,827 African Americans from 17 GWAS in the discovery stage, followed by replication in 11,544 African Americans and 47,117 Europeans [26••]. SNPs from three established loci (*TCF7L2*, *KCNQ1*, and *HMGA2*) and two novel loci (*HLA-B* and *INS-IGF2*) were significantly associated with T2D at a genome-wide level (Table 1).

This study highlights the opportunity and challenge of T2D loci discovery in African American populations. First, the associated SNPs at the two novel loci either have stronger effect (*HLA-B*) or have higher frequency (*INS-IGF2*) in individuals of African ancestry than in individuals of European ancestry, which led to higher study power to detect these associations. Similar observations also led to the novel discovery of *KCNQ1*, *UBE2E2*, and *C2CD4A-C2CD4B* in East Asians [41–43] and *SLC16A11* in Mexicans and Latin Americans [38]. It is intriguing that several of these variants confer T2D risk to specific populations only, as they are monomorphic or very rare in others.

Second, early GWAS arrays have substantially lower genome-wide coverage of common SNPs for African ancestry (28–49 %) compared to European ancestry populations (75–86 %) [44], leading to inefficient imputation and loss of power to detect association. Indeed, the HapMap imputed SNPs for the MEDIA study only cover 43.3 % of common SNPs in the 1000 Genomes ASW population at *r*^2^ ≥ 0.8, which partly explains the low number of genome-wide significant loci found in African Americans [26••]. Newer genotyping arrays that are population-specific or targeted to multiple ethnicities, such as the Axiom Genome-Wide PanAFR Array and the Infinium Multi-Ethnic Genotyping Array, have higher coverage for African ancestry populations, and thus will likely improve the power to identify African ancestry-associated variants. Improvement in the genome-wide coverage and haplotypes of diverse populations in reference panels for imputation such as the 1000 Genomes Project [45] and the Haplotype Reference consortium (http://www.haplotype-reference-consortium.org/) will also improve imputation quality, especially for identification of low frequency disease variants.

### Transferability and Fine Mapping of Established T2D Loci

Given the large number of loci associated with T2D and glycemic traits in European ancestry populations, it is of interest to test if these loci are also implicated in populations of different ancestry. Several strategies, including exact replication, local replication, and local transferability, have been used [44]. In this review, exact replication refers to testing association at the exact SNP identified from the discovery population with the same direction of effect. Local replication refers to testing association of all proxy SNPs in linkage disequilibrium (LD) with the original reported SNP, including itself. Local transferability refers to testing association of all SNPs, regardless of LD, in a region around the original reported SNP, bounded by a fixed distance or the most extended SNPs in LD [44]. Significant association is defined as *P* < 0.05 in exact replication. In local replication and local transferability, significant association is defined as *P* < 0.05 after adjustment for the number of effective SNPs in each locus. While an association in the local replication analysis implies that the causal variant(s) resides on the same haplotype between the discovery and follow up studies, the presence of association in the local transferability analysis may indicate either an independent new signal or represent the same association signal as in the original discovery study.

Early studies in African Americans and other populations focused on exact replication of the reported index SNPs. A study of 4045 African Americans demonstrated nominal replication of 5 out of 17 T2D index SNPs, with the strongest association observed at rs7903146 in *TCF7L2* (OR = 1.38, *P* = 1.25 × 10^−8^) [46]. Resequencing suggests that rs7903146 is the causal variant in this region [47], and this SNP remains the strongest association with T2D in African Americans [26••]. In the multi-ethnic study from the Population Architecture using Genomics and Epidemiology (PAGE) consortium, among 20 index SNPs from 18 established T2D loci, the direction of associations are largely consistent across populations of European American, African American, Hispanic, East Asian, American Indian, and Native Hawaiian ancestry [48]. However, risk allele frequencies of the index SNPs are substantially different at some loci, and only 7 index SNPs were nominally associated with T2D in 11,599 African Americans. Using genetic risk score to combine the effects of risk alleles of these index SNPs did not demonstrate conclusive association with T2D in African Americans [46, 49, 50].

Exact replication may produce false negative results when replicating in another population with different LD structure, particularly for African Americans given their greater genetic diversity and lower level of LD [51]. In a local transferability study of 7071 African Americans, 7 index SNPs from 40 T2D loci were replicated [52•]. Significant local transferability was observed for four loci, but only two of the local significant SNPs were in LD with the index SNPs from Europeans.

**Fig. 1:**
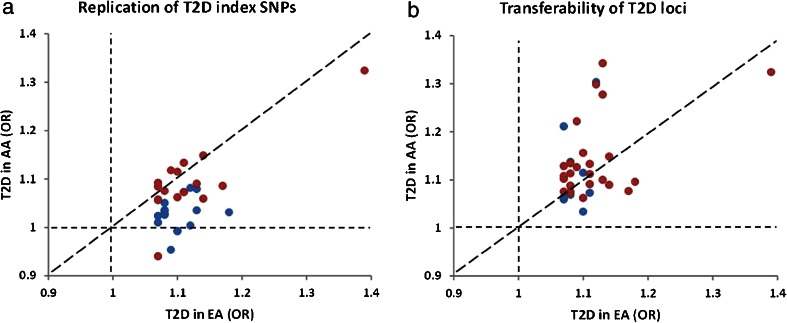
Replication and transferability of 34 genome-wide significant T2D loci in Europeans (*EA*) from the DIAGRAM consortium to African Americans (*AA*) from the MEDIA consortium. **a** odds ratio (*OR*) of risk alleles of index SNPs. **b** OR of index SNPs from Europeans and locus-wide lead SNPs from African Americans. *Red symbols* represent significant association at *P* < 0.05 in replication and *P*_locus_ < 0.05 in transferability analyses. *Blue symbols* represent insignificant associations

Among 65 established T2D loci reported by the DIAGRAM consortium, 34 index SNPs were genome-wide significant in Europeans [34]. By examining these 34 loci in the MEDIA consortium, exact replication only yielded 17 significant SNPs (Fig. 1a) whereas local transferability yielded 25 significant loci in African Americans (Fig. 1b) [26••]. In addition, among the lead SNPs at these 25 significant loci, only 12 lead SNPs were moderately correlated (*r*^2^ ≥ 0.3 in HapMap CEU population) with the European index SNPs. The results of this and other studies suggest that many, but not all, of the established T2D loci are shared across ancestries. The low level of exact replication is partly due to differential LD structure between Europeans and African Americans, where causal variant(s) may lie on different haplotypes. The possible presence of different or multiple causal variant(s) in different populations may account for the lack of correlation for some of the index SNPs between populations, although false positives in local transferability analysis could not be excluded.

Among genetic loci that are shared across different populations and display differential LD, the African American GWAS provides a great opportunity to fine map the associated regions through trans-ancestry meta-analysis [53]. While Europeans tend to have long range LD due to population bottleneck that facilitates gene discovery, it is difficult to distinguish which of the associated variants in high LD may be causal. Recent trans-ancestry meta-analysis of T2D GWAS in 110,452 individuals of European, East Asian, South Asian, Mexican, and Mexican American ancestry improved fine mapping resolution of European GWAS. Assuming a locus harbors a single causal variant, the number of SNPs and size of genomic interval that the causal variant has 99 % probability being included in the 99 % credible set was reduced in 9 of 10 established T2D loci in trans-ancestry meta-analysis [54••]. Future studies that include African Americans in trans-ancestry meta-analysis will maximize the resolution of fine mapping since African Americans tend to have lower and differential LD compared to other populations.

### Transferability and Fine Mapping of Established Glycemic Loci and their Associations with T2D in African Americans

As for glycemic traits, the PAGE consortium examined 7526 African Americans for exact replication of 9 fasting glucose and 2 fasting insulin index SNPs identified from European ancestry GWAS. Only three SNPs at the *MNTR1B* and *TCF7L2* loci were nominally associated with fasting glucose in African Americans [55]. Among 927 African Americans from the Howard University Family Study (HUFS), 3 of the 12 fasting glucose index SNPs at the *DGKB*, *SLC30A8*, and *TCF7L2* loci identified from the MAGIC consortium were directly replicated. Further local replication demonstrated additional associations at the *G6PC2*, *GCKR*, *MNTR1B*, and *IRS1* loci [56].

**Fig. 2:**
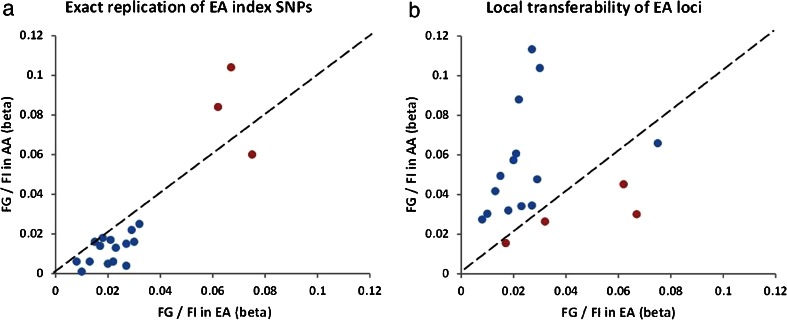
Replication and transferability of 18 fasting glucose (*FG*) and fasting insulin (*FI*) loci in Europeans (*EA*) from the MAGIC consortium to African Americans (*AA*) from the CARe study. **a** Effect size of risk alleles of index SNPs. **b** Effect size of index SNPs from Europeans and locus-wide lead SNPs from African Americans. *Red symbols* represent significant association at *P* < 0.05 in replication and *S*_locus_ < 0.05 in transferability analyses. *Blue symbols* represent insignificant associations

The Candidate Gene Association Resource (CARe) study examined 5984 African Americans in 16 fasting glucose and 2 fasting insulin loci reported by the MAGIC consortium [57•]. Exact replication yielded three SNPs at *GCK*, *MTNR1B*, and *G6PC2* for association with fasting glucose (Fig. 2a), with the strongest association observed at rs10830963 of the *MTNR1B* locus (*P* = 8.51 × 10^−9^). Local transferability demonstrated association of *GCK*, *MTNR1B*, and *FADS1* for fasting glucose, and *GCKR* for fasting insulin (Fig. 2b). Examination of the four lead locus-significant SNPs revealed only nominal association of the risk G allele of rs10830963 at *MTNR1B* for T2D risk in the MEDIA consortium (OR = 1.11, *P* = 0.02) [26••]. Taken together, the low level of transferability of glycemic trait loci between populations of European and African ancestry is consistent with findings for T2D. The lack of overlap of loci and correlation of their respective effect sizes between T2D and glycemic trait loci are also consistent with findings from the DIAGRAM and MAGIC consortia, which suggests that genes influencing normal physiological levels of metabolic traits may be different from those leading to pathological levels of metabolic traits that define T2D [58]. Given the sample size of the CARe study has limited power, the current effort of meta-analysis and fine mapping of glycemic traits from GWAS of African ancestry at the AAGILE consortium will provide a clearer picture for these questions.

### Association of Low Frequency Variants with T2D and Glycemic Traits

The development of exome chip derived from large-scale exome sequence studies allows cost-effective genotyping of ∼250,000 variants, primarily low frequency protein-altering variants, in large number of samples. However, the coverage of low frequency coding variants are lower in non-European populations given the discovery of coding variants are primarily from European ancestry individuals. The Cohorts for Heart and Aging Research in Genomic Epidemiology (CHARGE) T2D-Glycemia Exome consortium examined 60,564 non-diabetic subjects of European (84 %) and African (16 %) ancestry, and 98,368 T2D cases and controls of European (75 %), African (14 %), Asian (5 %), and Hispanic (6 %) ancestry using exome chips [59••]. In the combined ancestry analyses, a low frequency nonsynonymous SNP at *GLP1R* was associated with improved glycemic traits and lower T2D risk. Gene-based analysis of low frequency protein-altering variants revealed association of *G6PC2* with fasting glucose level. The associations at *GLP1R* and *G6PC2* were likely contributed predominantly, if not all, by the European data in the CHARGE consortium, as the same associations were also observed in Europeans by the Type 2 Diabetes Genetic Exploration by Next-generation sequencing in multi-Ethnic Samples (T2D-GENES) and Genetics of Type 2 Diabetes (GoT2D) consortia [60••]. On the other hand, analysis of established T2D loci in individuals of African ancestry revealed strong protective or risk effects on T2D for the minor alleles of low frequency nonsynonymous variants at *SLC30A8*, *ARAP1*, and *GIPR* (OR = 0.45–1.69, *P* < 1 × 10^−4^). These data support the contribution of low frequency variants and their allelic and locus heterogeneity on T2D risk and modulation of glycemic levels.

### Admixture Mapping

The African American population is admixed, with an average of 80 % African and 20 % European ancestry, although level of admixture varies considerably among individuals [61]. Given the higher prevalence of T2D in African Americans than in Europeans, it is hypothesized that some disease-associated genetic variants may differ substantially between the ancestral populations of African Americans and contribute to the disparity of disease prevalence. Admixture mapping typically uses ancestry informative markers to identify chromosomal regions containing risk alleles of a disease locus that are overrepresented in the ancestry with higher disease prevalence [62] (e.g., African ancestry for T2D). Admixture mapping has more power than linkage mapping when detecting disease-associated variants with highly differentiated frequency across ancestries. In the study by Cheng et al. [63], African ancestry was associated with 33 % increased risk of developing T2D between individuals with the highest and lowest tertiles of African ancestry. Subsequent admixture mapping in 7021 African Americans T2D cases and controls identified two potential loci at chromosomes 12p and 13q. However, another study did not show significant difference in the distribution of African ancestry between T2D cases and controls, while admixture mapping identified a potential signal on chromosome 11q [64]. Whether there is enrichment of risk alleles at disease loci in African ancestral haplotypes that may influence genetic risk for T2D remains to be determined.

### Population Differentiation and Selection at T2D Loci

Several population genetics studies have attempted to understand if population differentiation of T2D risk alleles is present and identify the respective factors that may contribute to T2D disparity across populations. While the risk alleles and their effect sizes are mostly consistent across different populations, strong differentiation of some T2D risk alleles and their loci have been reported, especially in the sub-Saharan African and East Asian populations [65, 66], despite not all loci demonstrating population differentiation [67•]. Most T2D risk alleles tend to show decreasing frequency along with human migration out of Africa, such that the African and East Asian populations carry the highest and lowest T2D genetic risk scores, respectively [66]. Genetic drift, migration history, and natural selection have been proposed to explain these differences [65, 66, 68]. The thrifty gene hypothesis proposed by Neel [69] suggests that genetic variants that promote efficient energy absorption and storage at time of feast and famine during the hunter-gatherer environment in the past leads to excess energy storage and increase risk for T2D in modern times with sedentary lifestyle and abundant food supply. However, recent positive selection was found on both risk and protective alleles at some loci [65, 67•], which is inconsistent with the thrifty gene hypothesis. There are, however, major drawbacks that may bias the interpretation of these studies. Most of the risk alleles and index SNPs are derived from European ancestry GWAS where causal variants are not directly tested. In addition, not all T2D loci found in Europeans are transferable to all populations. The identification of causal variants, both common and rare, through trans-ancestry fine mapping and sequencing in different populations will help to understand the genetic contribution to the disparity of T2D prevalence.

## Conclusions

Mapping genes for T2D and glycemic traits in African Americans has been challenging given the admixed nature of the population and the low degree of LD across the genome. Large-scale GWAS are ongoing and have shown some success in identifying novel genetic variants and demonstrated that substantial numbers of T2D-related loci are shared between Europeans and African Americans. The differential and lower level of LD in African Americans will likely greatly improve the fine mapping of causal variants in ongoing trans-ancestry meta-analysis efforts. On the other hand, far fewer genome-wide significant loci have been identified in GWAS of African Americans as compared to Europeans which could not be solely explained by the smaller sample size in African Americans, and a large proportion of missing heritability remains to be explained. Studies using next-generation sequencing in the genome, exome, and targeted regions are emerging in different populations including those of African ancestry. In particular, whole genome sequencing enables the generation of better reference panels to improve imputation quality and thus improve power of subsequent association studies. Additional large-scale genotyping of variants identified from sequencing studies is necessary to delineate the effect of low frequency and rare variants on T2D risk. Evidence of population differentiation and selection pressure on some T2D-related loci has been observed. Identification of loci and the respective causal variants in individual populations will be necessary to provide better insights as to whether genetic risks contribute to T2D disparity among different populations. Taken together, there is an urgent need for more genetic research in African ancestry populations to improve the understanding of genetic architecture of T2D and related traits.
